# Simian immunodeficiency virus-Vpx for improving integrase defective lentiviral vector-based vaccines

**DOI:** 10.1186/1742-4690-9-69

**Published:** 2012-08-22

**Authors:** Donatella RM Negri, Alessandra Rossi, Maria Blasi, Zuleika Michelini, Pasqualina Leone, Maria Vincenza Chiantore, Silvia Baroncelli, Gemma Perretta, Andrea Cimarelli, Mary E Klotman, Andrea Cara

**Affiliations:** 1 Department of Infectious, Parasitic and Immune-mediated Diseases, Viale Regina Elena 299, Rome 00161, Italy; 2 Department of Cell Biology and Neurosciences, Viale Regina Elena 299, Rome 00161, Italy; 3 Department of Therapeutic Research and Medicines Evaluation, Istituto Superiore di Sanità, Viale Regina Elena 299, Rome 00161, Italy; 4 Istituto di Biologia Cellulare e Neurobiologia-CNR, c/o ENEA-Casaccia, Roma 00123, Italy; 5 Department of Human Virology, Ecole Normale Supérieure de Lyon, Lyon, France; 6 Department of Medicine, Duke University Medical Center, Durham, NC, 27710, USA

**Keywords:** Lentiviral vector, Vpx, Vaccine, Dendritic cells, Integrase

## Abstract

**Background:**

Integrase defective lentiviral vectors (IDLV) represent a promising delivery system for immunization purposes. Human dendritic cells (DC) are the main cell types mediating the immune response and are readily transduced by IDLV, allowing effective triggering of *in vitro* expansion of antigen-specific primed CD8+ T cells. However, IDLV expression in transduced DC is at lower levels than those of the integrase (IN) competent counterpart, thus requiring further improvement of IDLV for future use in the clinic.

**Results:**

In this paper we show that the addition of simian immunodeficiency (SIV)-Vpx protein in the vector preparation greatly improves transduction of human and simian DC, but not of murine DC, thus increasing the ability of transduced DC to act as functional antigen presenting cells, in the absence of integrated vector sequences. Importantly, the presence of SIV-Vpx allows for using lower dose of input IDLV during *in vitro* transduction, thus further improving the IDLV safety profile.

**Conclusions:**

These results have significant implications for the development of IDLV-based vaccines.

## Background

The improvement of delivery systems in the vaccine and gene therapy fields is an important aspect to be considered in order to obtain an effective result. Successful use of Integrase (IN) defective lentiviral vectors (IDLV) for both immunization and gene therapy purposes has been reported by several groups [1-11]. IDLV are produced by incorporating a mutated form of the IN protein in the recombinant lentiviral particles, thus preventing the integration of the vector genome in the target cells and consequently improving on the safety profile of the parental integration competent lentiviral vectors. IDLV take advantage of the proficient expression of transgenes from the non-integrated forms of vector DNA, which are produced in the absence of integrated vector sequences.

Non-integrated DNA forms of IDLV have been shown to be long-lasting and transcriptionally active, both *in vitro* and *in vivo*, as long as the transduced cells are not dividing, even if at lower levels than those of the IN competent counterpart [12-14]. Recent reviews have described the progress made over the past few years and detailed the applications of IDLV [15-19]. From the standpoint of immunization, we demonstrated that a single inoculum with an IDLV expressing the human immunodeficiency type 1 (HIV-1) envelope protein in the mouse immunogenicity model was able to elicit strong and long lasting specific immune responses in the absence of vector integration, thus providing a safe and efficient delivery for vaccine purposes [9-11]. Importantly, several groups confirmed the use of IDLV as an effective vaccine delivery strategy [5-7], and we provided evidence that simian immunodeficiency (SIV)-based IDLV can be constructed and used as well for antigen delivery [10]. Concerning the potential use in a human setting, we recently demonstrated that IDLV-transduced human antigen presenting cells, such as monocyte-derived dendritic cells (DC) and macrophages, were able to induce antigen-specific T cells expansion of primed T cells *in vitro* using Influenza Matrix 1 protein (Flu-M1) as a model antigen [20].

Despite its potential, a limitation in the use of IDLV for a preventive vaccine is represented by the lower expression of the transgene compared to the IN competent counterpart. Indeed, although many vaccination strategies in small animals are successful, they may be less effective in larger animals, such as non-human primates or humans. In this setting, improving on the amount of the antigen delivered by IDLV is a critical issue, especially in the antigen presenting cells that play an essential role in the induction and expansion of vaccine-specific immune response. In this regard, Berger and colleagues demonstrated that SIV virus like particles (VLP) containing the SIV_MAC251_-Vpx protein greatly increased the transduction efficiency of IDLV in human DC and macrophages [21]. These results are in line with work from other groups, showing that Vpx of the SIV_SM_/HIV-2 lineage acts on cytoplasmic SAMHD1 protein, a HIV-1 restriction factor expressed in cells of the myeloid lineage that inhibits an early step of the viral life cycle, and demonstrating that SIV-Vpx induces proteasomal degradation of SAMHD1, ultimately enhancing HIV-1 infection in myeloid-lineage cells [22,23].

To exploit the use of SIV-Vpx in the context of IDLV-based vaccines, we evaluated whether IDLV containing SIV-Vpx (IDLV/Vpx) was more efficient than IDLV without Vpx in enabling functional expansion of primed antigen-specific CD8+ T cells. Results indicated that IDLV/Vpx expressing Flu-M1 was superior to IDLV alone in inducing *in vitro* expansion of primed Flu-M1-specific CD8+ T cells from PBMCs of Flu-M1 positive healthy donors, in the absence of integration. In addition, we show that SIV-Vpx did not improve the transduction efficiency of murine BM-derived DC, while significantly increased the transduction of simian DC, suggesting that the mouse model may not be appropriate to test an IDLV/Vpx based vaccine. We confirmed this hypothesis by immunizing mice with IDLV or IDLV/Vpx expressing HIV-Env and comparing at different time points the levels of immune responses induced.

## Results

### SIV-Vpx increases the transduction efficiency of IDLV in human DC

**Figure 1:**
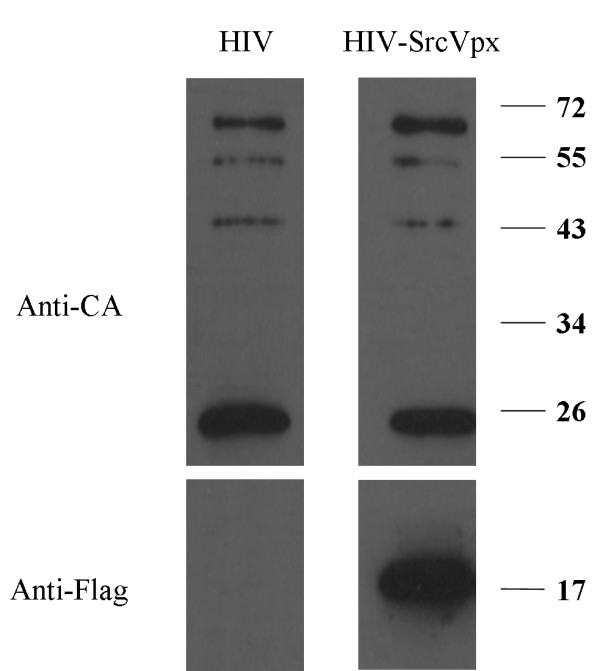
**Viral particles were produced by transient transfection of 293 T cells with (right) or without (left) plasmid expressing flagged SIV-Vpx.** Supernatants were harvested after 48 hrs and viral particles were purified onto a double-step sucrose gradient prior to WB analysis with the indicated antibodies. The positions of migration of molecular mass markers (in kilodaltons) are indicated on the right.

**Figure 2:**
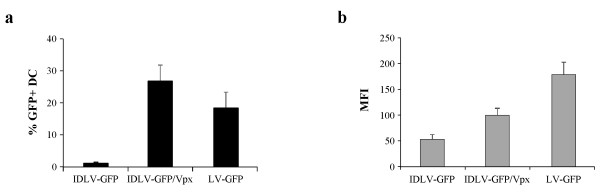
**Transduction of human DC with lentiviral vectors expressing GFP.** Human DC from different donors were transduced with IDLV-GFP, IDLV-GFP/Vpx or integrating LV-GFP (MOI 1). After 5 days from transduction the percentage of GFP-expressing DC (**a**) and the mean of fluorescence intensities (MFI) of GFP + cells (**b**) were measured. Results are expressed as average from eight different experiments. Error bars represent SD.

Presence of SIV-Vpx in viral particles was confirmed by Western blot analysis as described in Figure 1 and in the Methods section. Effect of SIV-Vpx on transduction efficiency of IDLV on human DC was evaluated on eight different donors using normalized amounts (MOI 1) of IDLV-GFP, IDLV-GFP/Vpx or IN competent lentiviral vector (LV-GFP) expressing GFP (Figure 2). GFP expression in transduced DC was evaluated in terms of percentage of GFP positive cells and mean of fluorescence intensity (MFI). At day 5 from infection, an average of 1.0% of GFP + DC was detected after infection with IDLV-GFP, while 26.5% and 18.9% of DC expressed GFP after transduction with the same amount of IDLV-GFP/Vpx or integrating LV-GFP, respectively (Figure 2a). These data indicated that inclusion of SIV-Vpx during vector preparation induced a statistically significant increment in the efficiency of transduction (IDLV/Vpx vs IDLV, P < 0.05), confirming data already shown by other groups using SIV-Vpx VLP [21]. As expected, MFI in DC transduced with the integrating LV-GFP was higher than MFI in DC transduced with IDLV-GFP or IDLV-GFP/Vpx (179 MFI vs 53 MFI and 100 MFI, respectively) (Figure 2b).

### SIV-Vpx improves the ability of IDLV-transduced human DC to induce expansion of M1-specific CD8+ T cells

In order to evaluate if inclusion of SIV-Vpx in the vector preparation could influence the functionality of human DC, their ability to expand antigen specific CD8+ T cells after IDLV transduction was analyzed. For this we used the Flu-M1 model as previously described [20]. A total of four selected Flu-M1 positive donors were used showing a percentage of M1-pentamer positivity within gated peripheral CD8+ T lymphocytes ranging from 0.15% to 0.34% (data not shown). These numbers represent baseline values of M1-reactive CD8+ T cells present in healthy donors that previously encountered influenza virus. DC from Flu-M1 positive donors were transduced with equal amount of vectors expressing influenza M1 protein, including IDLV-M1, IDLV-M1/Vpx, or integrating LV-M1. Vectors were constructed as already described [20] and used as indicated in Materials and Methods. DC transduced with vectors expressing M1 or control vector expressing GFP (IDLV-GFP/Vpx) were co-cultured with CD14-depleted autologous PBMC for 10 days. As controls, mature DC pulsed with M1-specific peptide (GIL) or left untreated were co-cultured with autologous cells, as above. The presence and expansion of M1-specific CD8+ T cells was evaluated by pentamer staining, and the functional activity of these cells was analyzed by IFN-γ production in the ELISPOT assay.

**Figure 3:**
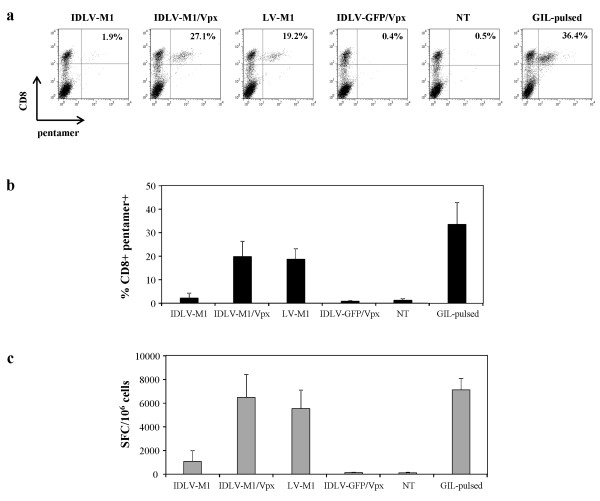
**Evaluation of M1-specific CD8+ T cell expansion by using transduced DC as antigen presenting cells.** DC were transduced with IDLV-M1, IDLV-M1/Vpx, integrating LV-M1 or IDLV-GFP/Vpx as an unrelated antigen (MOI 1). M1-specific peptide-pulsed (GIL-pulsed) or unpulsed DC (NT) were used as positive and negative controls, respectively. For pentamer assay (**a** and **b**), cells were stained with anti-CD8 PE-Cy5 antibody and PE-labeled HLA-A*0201 pentamer presenting the influenza matrix M1 epitope. The percentage of pentamer + cells was calculated within CD8+ T cells and in (**a**) a representative experiment is shown. (**b**) Percentages of CD8+ pentamer + T cells evaluated in four different healthy donors are indicated. Graphs show means ± SD. (**c**) The functionality of expanded CD8+ T cells was evaluated by IFN-γ-ELISPOT in the presence of M1 specific peptide. Results are expressed as spot forming cells (SFC) per 1x10^6^ cells.

In a representative experiment shown in Figure 3a, DC transduced with low levels of any M1-vector were able to expand M1-specific T cells. In particular, Figure 3b shows that while DC transduced with IDLV-M1 were able to induce a detectable expansion of M1-specific CD8+ T cells, DC transduced with IDLV-M1/Vpx induced higher significant expansion of M1-pentamer positive cells (2.1% and 19.7%, respectively; P < 0.05). Importantly, DC transduced with IDLV-M1/Vpx were able to induce an expansion of antigen specific CD8+ T cells similar to that observed in the co-culture with integrating LV-M1-transduced DC (18.6%; P < 0.05 compared to IDLV-M1 and IDLV-M1/Vpx). Untreated DC, as well as DC infected with control IDLV expressing GFP as an unrelated antigen, did not induce significant expansion of M1-specific CD8+ T cells. DC pulsed with intra-assay positive control GIL peptide showed the highest expansion of antigen-specific T cells (32.2%).

### Antigen-specific CD8+ T cells expanded by IDLV-M1/Vpx-transduced DC are functional

Functional analysis of M1-specific CD8+ T cells was performed by IFN-γ ELISPOT (Figure 3c). The expanded CD8+ T cells derived from the stimulation of CD14-depleted PBMC with IDLV-M1 transduced DC were able to produce IFN-γ in the presence of GIL peptide (average of 1086 SFC/10^6^ cells). As expected, the number of IFN-γ producing cells was higher in lymphocytes stimulated by DC transduced with IN competent LV-M1 (5500 SFC/10^6^ cells; P < 0.05). Of note, IDLV-M1/Vpx transduced DC induced functional CD8+ T cells showing a strong improvement in the number of SFC (6500 SFC/10^6^ cells) which was significantly higher than IDLV-M1 (P < 0.05) and similar to that observed when using IN competent LV-M1 (P > 0.05). The positive control represented by DC pulsed with M1-specific peptide showed similar results (7100 SFC/10^6^ cells). No significant IFN-γ production was detected in the case of co-culture with DC infected with IDLV-GFP or untreated DC (Figure 3c).

Overall these results indicate that IDLV-M1/Vpx-transduced DC efficiently enabled *in vitro* expansion of functional primed antigen specific CD8+ T cells. In particular, the increase in transduction efficiency in the presence of SIV-Vpx determined a strong and significant expansion of functional antigen-specific CD8+ T cells.

### Transduction with IDLV-M1/Vpx does not induce increase in DNA integration

**Figure 4:**
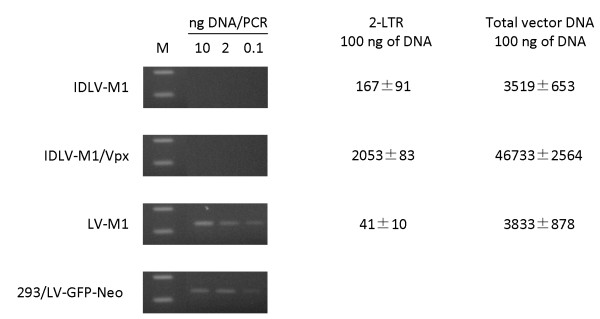
**Lentiviral vector sequences in human DC transduced with IDLV-M1, IDLV-M1/Vpx and LV-M1.** At day 7 from transduction DNA was extracted and used in semi-quantitative Alu-PCR for evaluating integrated vector sequences (left panels, a representative experiment out of three performed is shown). The 293 cell line stably transduced with the TY2-GFP-IRES-Neo vector (293/LV-GFP-Neo) was used as standard for evaluating integrated vector copies. PCR samples were run on a Ethidium bromide stained 2% agarose gel. Real-time DNA PCR was used for evaluating 2-LTR and late reverse transcription products copy number (right panel, average values of three independent experiments are shown), as described in Materials and Methods.

To evaluate if the increased transduction efficiency of DC with IDLV-M1/Vpx was due to increased vector integration, DNA recovered from DC transduced with IDLV-M1, IDLV-M1/Vpx and LV-M1 were used in a vector-specific Alu-PCR for the analysis of integrated vector sequences, as described in Materials and Methods. Only DC transduced with IN competent LV-M1 showed clear evidence of integrated vector sequences in as low as 0.1 ng of genomic DNA, while there was no indication of integration in IDLV-M1 and IDLV-M1/Vpx transduced DC in as high as 10 ng of genomic DNA (Figure 4, left panels). The 293 cell line stably transduced with a lentiviral vector expressing the Neomycin resistance gene (293/LV-Neo) was used as standard for evaluating presence of integrated vector sequences. Conversely, higher levels of late reverse transcription products and circular 2-LTR forms were found in DC transduced with IDLV-M1/Vpx than in DC transduced with IDLV-M1 or LV-M1 (Figure 4, right panel).

To further evaluate the effect of SIV-Vpx on the residual integration activity of IDLV, vectors expressing the neomycin resistance gene were generated with or without Vpx (IDLV-Neo/Vpx and IDLV-Neo, respectively) and used to transduce human 293 cells. IN competent LV-Neo was used as a reference positive control. Cells were infected with serial dilutions of each vector and subjected to selection with Geneticin, as described [9,10]. Results from two independent experiments showed that the number of colonies in the IDLV-Neo/Vpx-transduced Geneticin-selected cells was similar to that found in the IDLV-Neo-transduced Geneticin-selected cells (Table 1), indicating that SIV-Vpx did not alter the residual integration activity of IDLV. As expected, the number of colonies in the IN competent LV-Neo selected 293 cells was significantly greater (Table 1). At the indicated doses, IDLV-Neo and IDLV-Neo/Vpx integrated between 5.95 × 10^−4^ and 6.9 × 10^−4^ times less frequently than the IN competent vector, consistently with what has been found in other systems [9,10], demonstrating that IDLV maintained the integration deficient phenotype, regardless the presence of SIV-Vpx, with negligible amounts of residual integration activity.

**Table 1:** DNA recombination frequencies of IN defective vectors with and without Vpx and of IN competent control vector on 293 cell line

**Vector**	**Experiments**	**Number of colonies for 1x10**^**6**^**RT counts**	**Ratio Defective/competent**
IDLV-Neo	Exp1	129	6,6x10^-4^
	Exp2	99	6,3x10^-4^
IDLV-Neo/Vpx	Exp1	116	5,95x10^-4^
	Exp2	108	6,9x10^-4^
LV-Neo	Exp1	1,95x10^5^	-
	Exp2	1,56x10^5^	-

### Species-dependent susceptibility to IDLV/Vpx

**Figure 5:**
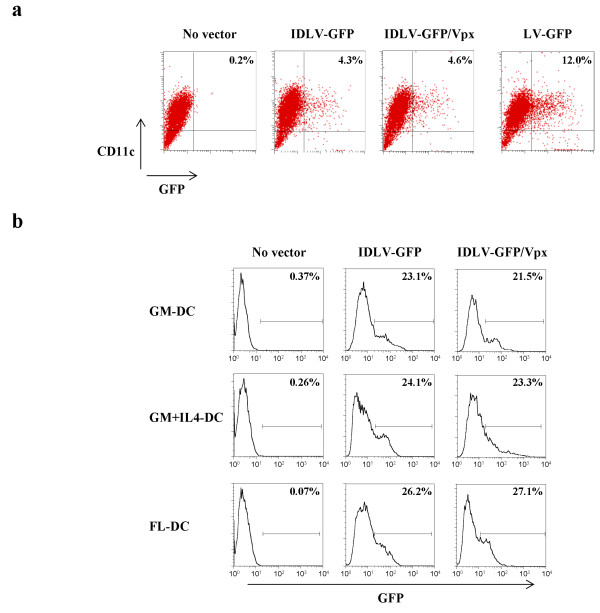
**Transduction of murine DC with lentiviral vectors expressing GFP.** (**a**) Murine bone marrow-derived dendritic cells (BMDC) differentiated with GM-CSF (GM-DC) were transduced with normalized amounts of IDLV-GFP, IDLV-GFP/Vpx or LV-GFP (MOI 1) or left untreated (no vector). After 5 days from transduction cells were analysed by FACS to evaluate the percentage of GFP expressing cells. A representative experiment out of three performed is shown. The percentages of GFP-expressing CD11c + DC are indicated within the dot plots. (**b**) Murine BMDC differentiated in the presence of GM-CSF alone (GM-DC), GM-CSF and IL-4 (GM+IL4-DC) or Flt3L alone (FL-DC) were transduced with normalized amounts of IDLV-GFP, IDLVV-GFP/Vpx (MOI 4) or left untreated (no vector). The expression of GFP was analyzed on CD11c + population. Percentages of GFP expressing cells are indicated within the histograms. A representative experiment out of three performed is shown.

The efficiency of IDLV in inducing a broad and long lasting immune response in murine models has been already established [18,19]. In order to evaluate if the mouse model of immunization could represent a suitable model for studying the *in vivo* effectiveness of a IDLV/Vpx based vaccine, bone marrow (BM)-derived DC from naïve Balb/c mice were transduced with IDLV-GFP/Vpx and compared to IDLV or LV expressing GFP. As shown by a representative experiment depicted in Figure 5a, the percentage of GFP + DC was similar, around 4%, in both IDLV and IDLV/Vpx transduced CD11+ DC. These results suggest that the presence of SIV-Vpx in the IDLV preparation did not improve murine DC transduction and that the efficacy of a vaccine based on IDLV/Vpx should not be tested in mice. It has been shown that BM-derived cells differentiated *in vitro* by using different cytokines and conditions generate DC with different morphology and characteristics [24]. To confirm the data obtained using GM-CSF differentiated DC (GM-DC), BM-derived cells were differentiated also with GM-CSF and IL-4 (GM + IL4-DC) or *fms* like tyrosine kinase 3 ligand (Flt3L) (FL-DC) cytokines. All these DC were transduced with IDLV-GFP or IDLV-GFP/Vpx at 4 MOI and the GFP expression analyzed after 5 days. A higher MOI was selected in order to verify if SIV/Vpx could be more active in a less sensitive model, such as murine cells. As shown in Figure 5b, SIV-Vpx did not generate any improvement in the transduction efficiency of IDLV. We also analyzed the MFI on GFP + murine DC from different experiments and results indicated that IDLV-GFP/Vpx transduced DC did not show significantly higher MFI than IDLV-GFP transduced ones (data not shown). We seldom found a minor population of transduced cells with higher MFI (less than 2%), regardless of the presence of Vpx or of the DC-differentiating cytokines. This is likely due to experimental variability when using primary cells treated with different stimuli.

**Figure 6:**
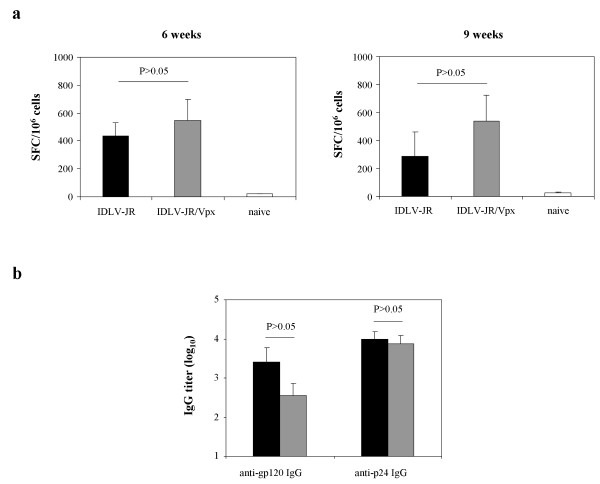
**Immune response in immunized mice.** (**a**) HIV-1-_JR-FL_ envelope T-cell responses measured by the IFN-γ ELISPOT assay on peripheral blood cells from mice immunized with IDLV-JR (black bars), IDLV-JR/Vpx (grey bars) and naïve mice (empty bars) at 6 and 9 weeks from injection. Analysis was performed on blood cells stimulated overnight with H2-D^d^ restricted specific Envelope JR-9mer peptide, as described in the Methods section. IFN-γ producing T cells are expressed as SFC/10^6^ cells after background subtraction. The error bars indicate the standard deviation among mice of the same group, and P-values are indicated for intergroup comparisons. (**b**) Anti-gp120 and anti-p24 IgG measured by ELISA in plasma samples of mice immunized with IDLV-JR (black bars) or IDLV-JR/Vpx (grey bars) at 6 weeks after immunization. Plasma samples from immunized mice were analyzed separately. Results are expressed as the IgG mean titer of 4 mice per group. Error bars indicate the standard deviations amongst mice of the same group. P-values are indicated for intergroup comparisons.

Despite these data, an *in vivo* immunization in mice was performed. HIV-gp120 was used as an antigen in order to evaluate both cellular and humoral antigen-specific immune response after a single immunization [9]. As shown in Figure 6a, the induction of gp120 specific CD8+ T cells was similar in mice immunized with IDLV-JR or IDLV-JR/Vpx at both 6 weeks after immunization (437 and 546 SFC/10^6^, respectively; P > 0.05) and later on at 9 weeks after injection (289 and 537 SFC/10^6^, respectively; P > 0.05). The presence of anti-gp120 antibodies was evaluated at 6 weeks after immunization in plasma of immunized mice by ELISA (Figure 6b). As expected, the immunization with IDLV did not induce high level of antibodies as already observed in other studies [9,25]. Of note the immunization with IDLV-JR/Vpx did not improve humoral response. To verify that the mice in both groups had been injected with the same amount of IDLV vectors, anti-HIV Gag antibody response was evaluated. Indeed, the injection of lentiviral vectors induces antibodies directed versus the gag protein present in the viral particles [25,26]. As shown in the Figure 6b, anti-p24 antibodies were similar in plasma from both groups of mice, confirming that the two groups received similar amounts of vectors. All together these results suggest that the mouse model is not suitable for the validation of IDLV/Vpx -based vaccine.

**Figure 7:**
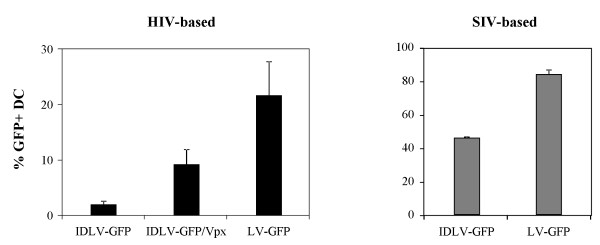
**Transduction of simian DC with lentiviral vectors expressing GFP.** Simian monocyte-derived DC from different monkeys were transduced with equal amounts (MOI 1) of HIV-based IDLV-GFP, IDLV-GFP/Vpx, LV-GFP (left panel) or with SIV-based IDLV-GFP or LV-GFP (right panel). After 5 days from transduction cells were analysed by FACS to evaluate the percentage of GFP expressing cells. Results are expressed as average from eight different experiments. Error bars represent SD.

Non-human primates (NHP) represent an important model for evaluating vaccine approaches against many infectious diseases such as HIV-1, malaria and tuberculosis. To evaluate if the use of IDLV/Vpx might be advantageous in NHP, simian DC derived from 4 rhesus and 4 cynomolgus monkeys were transduced with GFP-expressing IDLV with or without SIV-Vpx. As controls, we used SIV-based IDLV and LV expressing GFP. This was done in order to evaluate differences in transduction ability among the HIV- and SIV-based vectors in simian cells. As shown in Figure 7, IDLV-GFP/Vpx showed a significant increase in the percentage of transduced DC, compared to IDLV-GFP (9.3% vs 2.5%; P < 0.05). The integrating LV counterpart showed higher percentage than that observed in both IDLV (21.7%; P < 0.05 compared to both IDLV-GFP and IDLV-GFP/Vpx). Compared to human DC, simian DC were less susceptible to IDLV-GFP/Vpx transduction. This is likely due to the presence of species-specific restriction factors, as it has been previously shown using HIV-1 in simian cells [27,28]. As expected, SIV-based IDLV and LV were more efficient in transducing simian DC than the corresponding HIV-based vectors (Figure 7, left panel) at the same low MOI (46% and 84%, for the SIV-based IN defective and IN competent vectors, respectively). These results suggest that SIV-based IDLV, compared to HIV-based IDLV/Vpx, represent a better vector for testing the efficacy of IDLV-based vaccine in a relevant NHP animal model.

## Discussion

In the present study we demonstrate that IDLV/Vpx efficiently transduce human monocyte-derived DC, leading to an improvement of the functional properties of IDLV, as evaluated by *in vitro* expansion of antigen-specific CD8+ T cells. This is important since DC represent a major cell type mediating the immune response and recent work has shown that vaccines delivered by IDLV targeting DC induce strong antigen-specific immunity [7]. A recent report showed that non-infectious SIV_MAC251_ VLP carrying Vpx efficiently improved transduction of human antigen presenting cells with IDLV [21]. To evaluate a future use of this vector for vaccine purposes, the functional ability of DC to expand an antigen-specific response after transduction with IDLV/Vpx was analyzed. We opted for a lower input virus than previously reported [20] in order to further increase the safety level of the vaccine and to better discriminate between IDLV and IDLV/Vpx functional ability. The transduction efficiency of IDLV/Vpx was compared with IDLV alone or with integrating LV, and we showed that IDLV/Vpx was significantly more efficient than IDLV alone and similar to integrating LV. Our results are consistent with those published by Berger *et al*. and could be explained by recent papers showing that SIV-Vpx interacts with SAMHD1, a HIV-1 restriction factor in myeloid cells, such as DC and macrophages, that inhibits an early step of the viral life cycle [21,22]. It has been demonstrated that SIV-Vpx relieves the inhibition of lentivirus infection leading to highly efficient proteasome-dependent degradation of the protein SAMHD1 [23]. Consequently, through the action of Vpx, IDLV becomes “fully competent” reaching high levels of transduction, with expression levels approaching those observed with integrating LV.

We also demonstrated that the percentage of functional antigen-specific CD8+ T cells recovered after co-culture with IDLV/Vpx transduced DC was significantly higher than that observed in Vpx-less IDLV-transduced DC co-cultures, and similar to that of integrating LV-transduced DC. To ascertain that the increased expression in IDLV/Vpx-transduced DC was not caused by an increase in DNA integration, PCR and real time PCR analyses on transduced DC were performed. Our results demonstrated that only the late reverse transcription products and the 2-LTR circular forms were increased in the IDLV/Vpx transduced DC, in the absence of detectable integrated vector sequences. This suggests that the higher transduction efficiency of IDLV/Vpx is due to a more efficient reverse transcription culminating with a higher number of transcriptionally active episomal forms of vector DNA in the absence of integration, as previously reported in similar systems [21]. Importantly, recent reports [29,30] have shown that SAMHD1 in dendritic cells is a deoxynucleoside triphosphate triphosphohydrolase which restricts the replication of HIV-1 by depleting the intracellular pool of dNTP. This would decrease HIV-1 DNA synthesis rate ultimately slowing reverse transcription and impairing viral replication. While it is likely that the effect of Vpx is not specifically on reverse transcription, the Vpx-induced degradation of SAMHD1 results in a larger intracellular dNTP pool which allows reverse transcription to proceed and restores permissiveness of the cells to infection.

The efficacy of IDLV as a delivery system for immunization purposes *in vivo* has been demonstrated in mice by our group and other groups in different model systems [18,19]. It is now firmly established that in mice IDLV-vectored antigens are able to induce sustained immune response over time, which is protective against challenge with wild-type virus and tumor cell lines expressing the model antigen. To verify the ability of SIV-Vpx to increase the transduction efficiency of immune cells and consequently to induce an improved immune response in the mouse model, we firstly evaluated the transduction efficiency of GFP-expressing IDLV/Vpx on murine BM-derived DC, in comparison with that of IDLV alone. Our results indicated that SIV-Vpx did not improve on the transduction efficiency of murine DC differentiated either with GM-CSF alone or GM-CSF and IL4 or Flt3L, suggesting that mice do not represent a proper model to evaluate an IDLV/Vpx-based vaccine strategy. To further verify the suitability of the mouse model, we immunized mice with IDLV or IDLV/Vpx and the data confirmed the results obtained *in vitro*. One explanation could be that the murine analogue of SAMHD1, the mouse IFN-γ induced gene (MG11) which presents only 72% identity with SAMHD1 [31], is not properly targeted by SIV-Vpx. Indeed, a recent report [30] showed that while Vpx counteracted human and rhesus SAMHD1, it was not active against the mouse homolog of SAMHD1. In particular, Vpx prevented the SAMHD1-mediated decrease in dNTP concentration by inducing degradation of human and rhesus macaque SAMHD1, but had no effect on mouse SAMHD1.

Conversely, based on SIV-Vpx origin, the most appropriate animal model to test IDLV/Vpx vaccine should be the NHP. Our data showed that IDLV/Vpx was significantly more efficient in transducing simian DC compared to IDLV alone, but less efficient than integrating LV. The different susceptibility of simian DC compared with human DC to HIV-1 infection has been already established [27,28,32,33]. Bypassing the restriction factor counteracted by Vpx could not be sufficient for HIV-based IDLV to become fully competent in simian DC. Other species specific restrictions could still interfere with the transduction efficiency, and this effect could be particularly evident at low viral input [27,28,32,33]. To overcome these issues and to set the optimal conditions for inducing a proficient immunization with an IDLV-based vaccine *in vivo*, two avenues might be taken under consideration. The first one aims at using SIV-based IDLV, in place of HIV-based IDLV, in monkeys, since SIV-based vectors not only intrinsically contain Vpx in the packaging vector, but also the full complement of viral proteins necessary for counteracting the specie-specific restriction factors present in simian DC [27,28,32,33]. Indeed, we show here that the efficiency of transduction of simian DC with SIV-IDLV was comparable to that observed in human DC transduced with IDLV/Vpx and higher than that observed with HIV-based IDLV and integrating LV in simian DC. We may thus speculate that immunization with a SIV-based IDLV in monkeys could mimic a vaccine delivered by HIV-based IDLV/Vpx in humans. The second option might consider the use of HIV-based IDLV/Vpx in a humanized mouse model, such as the humanized Bone marrow/Liver/Thymus (BLT) mice [34]. Humanized BLT mice have been shown to exhibit a complete, systemic and self-renewing reconstitution of the human hematopoietic lineages including T, B, monocyte, DC and natural killer cells, thus facilitating the induction and evaluation of functional human immune responses following immunization. In addition it has been reported that BLT mice have mucosal reconstitution with human immune cells which is competent for mucosal or systemic infection with replication competent HIV-1 virus [35], necessary for evaluating the outcome of a successful immunization. Recently, it has been proven that this model perfectly mimics human response to topical application of the tenofovir to prevent HIV-infection, as already seen in the human clinical trial CAPRISA 004 [36], further validating BLT mice as a good model in the HIV-preventive treatments [37]. To date, vaccine studies in BLT mice have not yet been performed, but this model seems to be eligible also for studying induction of immune responses by vaccine strategies. In conclusion, our data strongly suggest that IDLV/Vpx represent a new, efficient and safe delivery system useful for vaccine strategies, to be tested *in vivo* in a proper animal model system.

## Conclusions

These results indicate that inclusion of SIV-Vpx protein in IDLV preparations significantly improves the transduction of DC, thus increasing the ability of transduced DC to act as functional antigen presenting cells. Further, the presence of SIV-Vpx allows for using a lower amount of input IDLV during *in vitro* transduction, thus improving the safety profile of IDLV and the potency of the immune response with significant implications for the development of vaccines based on IDLV.

## Methods

### Vector construction

Transfer vectors expressing Flu-M1 (pTY2CMV-M1W) and GFP (pTY2CMV-GFPW) have been already described [20]. Transfer vector pTY2CMV-JRW, expressing the HIV-1_JR-FL_ envelope, was obtained by removal of the mGM-CSF cassette from plasmid pTY2CMV-JRmZ [9]. Transfer vector pTY2-GFP-IRES-Neo, which contains the Neomycin phosphotransferase resistance gene (Neo), was obtained by cloning a ClaI/SalI restricted fragment of DNA from pTY2CMV-GFP, containing the CMV promoter and the GFP sequence, and a SalI/EcoRV restricted fragment of DNA from plasmid pFB-Neo (Stratagene, Agilent Technologies, Inc., Santa Clara, CA), and containing the IRES2-Neo sequence, into the pTY2 ClaI/EcoRV backbone plasmid. The HIV-based packaging plasmids IN competent (pCMVdR8.2) and IN defective (pcHelp/IN-), the SIV-based packaging plasmids IN competent (pADSIV3+) and IN defective (pADSIVD64V), and the VSV.G envelope-expressing pMD.G plasmids have been already described [9,10,38]. For construction of SIV-Vpx expressing plasmid, Vpx derived from the SIV_MAC251_ strain was modified by fusing it to a heterologous membrane targeting domain derived from c-Src and to a FLAG epitope, into the plasmid pEF/myc/cyto (Gibco Invitrogen, Carlsbad, CA). This modification allows high levels of incorporation of SIV-Vpx into HIV-1 virus particles (VLP) and will be presented in more details elsewhere (Cimarelli et al., manuscript submitted). Expression of SIV-Vpx and incorporation into HIV-1 VLP was verified by Western blot using a polyclonal anti-FLAG antibody (Sigma-Aldrich Chimie S.a.r.l., Lyon, France) while presence of Gag was evaluated with an anti-Gag polyclonal antibody (NIH Repository Reagents, Cat. #4250), as primary antibodies. Secondary antibodies included an anti-rabbit HRP-conjugated IgG (Dako, Dako France S.A.S.). The immunocomplexes were visualized using chemiluminescence ECL detection system (Super Signal West Dura Extended Duration Substrate- PIERCE/ Thermo Scientific, USA).

### Production of recombinant vectors

The human epithelium kidney 293 T cell line was maintained in Dulbecco's Modified Eagle’s medium (Euroclone, Life Sciences Division, Pero, Milan, Italy) supplemented with 10% fetal bovine serum (FBS) (Euroclone) and 100 units/ml of penicillin-streptomicin-glutamine (PSG) (Gibco Invitrogen, Paisley, UK). For production of recombinant lentiviral vectors IN defective (IDLV) and IN competent (LV) expressing GFP (IDLV-GFP, IDLV-GFP/Vpx and LV/GFP), M1 (IDLV-M1, IDLV-M1/Vpx and LV/M1), HIV-1 gp120_JR-FL_ (IDLV-JR and IDLV-JR/Vpx) or Neomycin resistance gene (IDLV-Neo, IDLV-Neo/Vpx and LV-Neo), 293 T cells were transiently transfected on 10 cm Petri dishes using the Calcium Phosphate-based Profection Mammalian Transfection System (Promega Corporation, Madison WI, USA) as previously described [9,10]. A total of 12 μg of plasmid DNA were used for each plate in a ratio 6:4:2 (transfer vector: packaging vector: VSV.G vector). For the preparation of IDLV containing SIV-Vpx and expressing GFP or Flu-M1 (IDLV-GFP/Vpx and IDLV-M1/Vpx, respectively), 10 μg of SIV-Vpx codifying plasmid were included in the transfection. Recombinant IDLV and LV were produced using pcHelp/IN- and pCMVdR8.2 packaging plasmids, respectively, if HIV-based, or pADSIVD64V and pADSIV3+, if SIV-based. After 48 h, cell culture supernatants were recovered, cleared from cellular debris and passed through a 0.45 μM pore size filter (Millipore Corporation, Billerica, MA, USA). Concentration vectors containing supernatants were ultracentrifuged (Beckman Coulter, Inc., Fullerton, CA, USA) on a 20% sucrose gradient (Sigma Chemical Co. St. Louis, MO, USA) and viral pellets were resuspended in 1X PBS. Viral titers for the GFP-coding vectors were normalised by exogenous reverse transcriptase (RT) activity assay [39] and titration on 293 T cells [9,40]. For the Flu-M1 and the HIV-1_JR-FL_ Envelope-coding IDLV and LV, titration was performed by the RT activity assay over standards of known infectivity and the vector-associated RT activity was compared with the one of IDLV-GFP or LV-GFP-coding virions of known infectious titers, thus allowing for the determination of their infectious titer units [40].

### Generation of primary human and simian monocyte-derived DC and transduction with lentiviral vectors

Human cells were obtained from healthy blood donor volunteers. Healthy Macaca *mulatta* (rhesus monkey) and Macaca *fascicularis* (cynomolgous monkey) were housed in the non-human primate facility at the Ente Nazionale Energia Alternativa (ENEA) in Casaccia (Rome, Italy). The animals were kept according to international, European and Italian guidelines.

Human and simian peripheral blood mononuclear cells (PBMC) were isolated by density gradient centrifugation using Lympholyte (Cedarlane Laboratories Ltd.,Burlington, NC, USA). PBMC were incubated with anti-human CD14 microbeads (Miltenyi Biotec, Calderara di Reno, Bologna, Italy) following manufacturer instructions. Purified monocytes were resuspended in medium RPMI 1640, containing 10% FBS (Euroclone), 100 units/ml of PSG (Gibco Invitrogen), non-essential amino acids (Gibco Invitrogen), sodium pyruvate 1 mM (Euroclone) and HEPES buffer solution 25 mM (Euroclone). Monocytes were seeded in 24 well plate at 1x10^6^ per well in 1 ml in the presence of 50 ng/ml GM-CSF (Immunological Sciences, Rome, Italy) and 35 ng/ml of IL-4 (Immunological Sciences) to differentiate monocytes in DC. On day 5, 0.25x10^6^ DC were infected with 1 multiplicity of infection (MOI) of viral supernatant. The cells were centrifuged for 1 hour at 1500 rpm. After 2 hrs of incubation at 37°C, cells were washed once and seeded in complete medium supplied with cytokines. After 5 days from transduction with lentiviral vectors expressing GFP, the expression of GFP was analysed by FACScalibur (BD Biosciences, San Jose, CA, USA).

### Generation of murine bone marrow-derived DC and transduction with lentiviral vectors

Bone marrow (BM) was recovered from Balb/c mice, as described [41]. Briefly, BM cells were obtained from tibiae by syringe insertion into one end of the bone and flushing with RPMI medium. For DC generation, cells from BM were suspended at 1x10^6^ cells/ml in complete medium containing 50 μM 2- mercaptoethanol (Sigma Chemicals, Co., St. Louis, MO, USA) and 10 ng/mL of rmGM-CSF (Peprotech, Rocky Hill, NJ, USA), 10 ng/ml recombinant mouse GM-CSF and 20 ng/ml IL-4 (Peprotech) or 40 ng/ml recombinant mouse Flt3L (R&D Systems, Inc., Minneapolis, MN, USA), to differentiate GM-DC, GM + IL4-DC or FL-DC, respectively. GM-CSF and IL-4 were added in fresh medium each 3 days. At 7 days, loosely adherent cells were then collected, analyzed for CD11c expression and transduced with lentiviral vectors expressing GFP, as above described. After 5 days, BMDC were surface stained for CD11c FITC (BD Biosciences) and analyzed by FACScalibur for the expression of GFP.

### *In vitro* expansion of human antigen-specific CD8+ T cells

Human donors were selected based on the presence of CD8+ T cells specific for the HLA-A*0201-restricted M1 epitope (GILGFVFTL), as described [20]. PE-labeled HLA-A*0201 pentamer presenting the influenza matrix M1 epitope (aa 58–66) (M1-pentamer) and control PE-labeled HLA-A*0201 pentamer presenting the HIV-gag peptide (SLY-pentamer) were provided by Proimmune (The Magdalen Centre, Oxford Science, Oxford, U.K.). PBMC (2x10^6^) were washed in wash buffer (0.1% BSA in PBS), spun down and resuspended in residual liquid. Either M1- or SLY-pentamer (2 μl) was added to the cells, incubated at 4°C for 20 minutes and further incubated for 15 min on ice with PE-Cy5-labeled anti-CD8 monoclonal antibody (Immunological Sciences). After washing, cells were resuspended in 1% formaldehyde in PBS and CD8+/pentamer + cells were analyzed on FACSCalibur (BD Biosciences) using CellQuest software. Monocytes from M1-positive selected donors were isolated, differentiated into DC and transduced with lentiviral vectors expressing M1, as above described. Maturation of DC was induced by adding lipopolysaccharide (LPS, 0.5 μg/ml; Sigma-Aldrich) to the medium on day 7, and on day 8 mature DC were used for stimulation of autologous PBMC. In particular, DCs transduced with lentiviral vectors expressing M1 protein, GFP or left untreated were co-cultured with autologous PBMC depleted of CD14 cells, at an effector (T cell)-to-stimulator (APC) ratio of 10:1 for 10 days in the presence of IL-2 (50U/ml; BD Biosciences) and IL-7 (5 ng/ml; Thermo Scientific, Rockford, IL).

### Analysis of M1-specific CD8+ T cells by pentamer staining and IFN-γ ELISPOT

The expansion of M1-specific CD8+ T cells was evaluated by using PE-labeled M1-pentamer, as described above. The IFN-γ ELISPOT assay was performed by using reagents from BD Biosciences. The 9mer containing the HLA-A*0201 restricted M1 epitope (GILGFVFTL) (Primm s.r.l. San Raffaele Biomedical Science Park, Milan, Italy) and the HLA-A*0201 restricted HIV-1 gag 9mer peptide (SLYNTVATL) (Primm s.r.l.) were used at 1 μg/ml as specific and unrelated stimuli, respectively. Medium alone and PHA (Sigma, Chemical Co. St. Louis, MO, USA) were used as negative and positive controls, respectively. Samples were scored positive when was present a minimum of 50 spots per 10^6^ cells and a fold of 2 or higher compared to the unrelated peptide.

### Residual integration activity of the IN defective vector

The human epithelium kidney 293 cell line was seeded at 5 x 10^4^ cells per well in 6-well plates. Next day cells were transduced with serial ten-fold dilutions of IN competent LV-GFP-IRES-Neo, IN defective IDLV-GFP-IRES-Neo and SIV-Vpx carrying IN defective IDLV-GFP-IRES-Neo/Vpx vectors (range 1 x 10^5^ to 1 x 10^1^ RT counts for the IN competent vector and 1 x 10^6^ to 1 x 10^2^ RT counts for IDLV). The medium was removed 24 hours later and replaced with medium supplemented with 800 μg of Geneticin (Gibco Invitrogen) every 3 days. Cells were grown for two weeks, and developed clones were fixed with methanol and stained with Giemsa (Sigma). Clones on each well were counted and expressed as number of colonies/10^6^ RT counts. One separate well infected with TY2-GFP-IRES-Neo/IN + was allowed to grow under Geneticin pressure for production of 293/LV-GFP-Neo cell line, used as a positive control for integration in the vector sequence analysis.

### Vector sequences in human DC transduced with IDLV

DNA from DC was extracted using the mi-Tissue Genomic DNA Isolation Kit (Metabion International AG, Martinsried, Germany). Extracted DNA was quantified by determination of RNaseP content by Real-Time PCR using 20X RNaseP Primer-Probe (Vic) Mix (Applied Biosystems, Foster City, CA, USA). The integrated vector sequence was evaluated using a modified Alu-PCR assay. The 293 cell line stably transduced with the TY2-GFP-IRES-Neo vector (293/LV-GFP-Neo) was used as standard for evaluating integrated vector copies and absence of circular forms. In a first set of amplifications, two primers based on the human Alu repeat sequences, forward primer (AluS: 5′-TCCCAGCTACTGGGGAGGCTGAGG-3′), reverse primer (AluAs: 5’-GCCTCCCAAAGTGCTGGGATTACAG-3’) and one forward primer based on the CMV promoter sequence of the vector (CMVfor: 5′-ACGCCAATAGGGACTTTCCATTGAC-3′) (Eurofins MWG Operon, Ebersberg, Germany) were used to generate a mixture of sequences. Reactions were performed on 10, 2 and 0.1 ng of genomic DNA. Primers were used at a final concentration of 300nM for AluS/AS and 100 nM for CMV using 1X AmpliTaq Gold PCR Master Mix (Applied Biosystems). PCR conditions were 95°C, 5 min; 94°C, 10 s; 60°C, 10 s; 72°C, 4 min, 25 cycles of amplification. A nested PCR was performed on 1:100 dilution of the first PCR product using two internal primers in the vector genome (m902: 5'-AAAGGGACTGGAAGGGCTAATTCACT-3'; AA55: 5'-CTGCTAGAGATTTTCCACACTGAC-3') producing a 230 bp amplicon in the 3’LTR region. All primers were used at a final concentration of 300nM using 1X AmpliTaq Gold PCR Master Mix (Applied Biosystems). PCR conditions were: 95°C, 5 min; 94°C, 30 s; 60°C, 30 s; 72°C, 30 s, with a final extension step of 10 min at 72°C for 35 cycles of amplication in a 9700 Perkin-Elmer Thermocycler.

The quantification of 2-LTR (U3)–deleted circular forms (2-dLTR) was performed by a quantitative real-time PCR, using Syber Green I chemistry with 10, 5 and 2 ng of DNA and 900nM each of primers (477: 5′-GTGACTCTGGTAACTAGAGA-3′ and R485: 5′-AGAGAGCTCCCAGGCTCAG-3′) spanning the junction between the two U3-deleted (dU3) dLTR (U5–dU3). Amplifications were carried out in 25 μl of reaction volume, performed in duplicate with 2X TaqMan Universal master mix (Applied Biosystems). The kinetic PCR reaction conditions were 1 cycle at 50°C, 2 min; 1 cycle at 95°C, 10 min; 40 cycles at 95°C, 15 s, 60°C, 1 min on the ABI Prism 7500-FAST Real-Time PCR System (Applied Biosystems). The plasmid TA-2dLTR [9] was used to generate the standard curve (ranging from 10,000 to 1 copies) for 2-dLTR DNA circles quantification, and each point was performed in triplicate; fluorescent products were detected at the last step of each cycle. After amplification a melting curve was generated. HIV-1 and RNase P standard curves had slopes between −3.43 and −3.64 and the coefficients of correlation were >0.99. All samples and controls were run in duplicate and the normalized value of 2-dLTR copies was expressed as number of copies/100 ng of total DNA.

The quantification of total vector DNA was performed by a quantitative real-time PCR, using 10, 5 and 2 ng of DNA in 15 μl final volume. Primers were used at a final concentration of 500 nM while probe was 250 nM. Sequences were as follow: mZ902: 5'-ACTGGAAGGGCTAATTCACT-3'; ZAA55: 5'-GCTAGAGATTTTCCACACTGAC-3' and probe 5’-(6-FAM)-CCAGAGTCA-(ZEN)-CACAACAGACGGGCACA-3’(IABIkFQ) (IDT, Leuven, Belgium). A standard curve, derived from serial dilution of 293/LV-GFP-Neo cell line containing the target sequence and ranging from 1 to 10^5^ copies was measured in triplicate. Reaction mixtures contained 1x TaqMan Fast Universal PCR master mix (Applied Biosystems). After an initial incubation at 95°C for 2 minutes, 40 cycles of amplification were carried out as follows: denaturation for 3 s at 95°C, annealing for 30 s at 60°C, and extension for 30 s at 72°C. Reactions were carried out and analyzed using the ABI Prism 7500-FAST Real-Time PCR System (Applied Biosystems).

### Mice immunization

Mice were kept in accordance with the European Union guidelines and Italian legislation. All protocols were approved by the authors' Institutional Review Board. Six to eight weeks old BALB/c mice were injected once intramuscularly with 7.2 × 10^6^ RT units of either IDLV-JR or IDLV-JR/Vpx vectors formulated in 0.2 ml of PBS. Four mice were injected for each group and naïve animals were kept as negative control. Presence of HIV-Env-specific CD8+ T cells was evaluated at different time points after vaccination in blood. Mice were orbitally bled collecting 200 μl of whole blood in the presence of K-EDTA anticoagulant. Plasma were separated from cell fractions by low speed centrifugation and kept at −80°C for the measurement of anti-gp120 and anti-p24 antibodies, as already described [9,25]. Leukocytes, obtained after Ammonium Chloride Potassium (ACK) treatment, were counted, suspended in complete medium containing 50μM 2- mercaptoethanol and analyzed for the presence of antigen specific T cells by IFNγ ELISPOT (BD Biosciences), as already described [9] using H2-D^d^-restricted HIV-1 gp120 V3 loop epitope (IGPGRAFYT, Primm) at 2 μg/ml. The unrelated GFP 9mer epitope (HYLSTQSAL, Primm) and Concanavalin A (Sigma) were used as negative and positive controls, respectively. Samples were scored positive when a minimum of 50 spots per 10^6^ cells and a fold of 2 or higher was present compared to the unrelated peptide.

### Statistical analysis

Statistical analyses were performed by using Student’s t-test. All P values were considered significant if less than 0.05.

## Competing interests

The authors declare no competing financial interests.

## Author contributions

DRMN designed the experiments and wrote the paper. AR performed the immunological assays. MB performed the integration assay. ZM performed the PCR experiments. PL prepared the vector. MVC evaluated GFP expression. SB performed the statistical analysis. GP provided the Non Human Primate samples. AC prepared the Vpx-expressing vector. MEK discussed the experiments and wrote the paper. AC designed the experiments and wrote the paper. All authors read and approved the final manuscript.
